# A Rare Case of Peripheral Exophytic Osteoma of the Mandible Arising From an Extraction Site: Cone-Beam Computed Tomography (CBCT) Findings

**DOI:** 10.7759/cureus.64221

**Published:** 2024-07-10

**Authors:** Swapnil Mohod, Komal V Dadgal, Rajanikanth K, Suwarna Dangore-Khasbage, Aayushi Pakhale, Alka Hande

**Affiliations:** 1 Oral Medicine and Radiology, Sharad Pawar Dental College and Hospital, Datta Meghe Institute of Higher Education and Research, Wardha, IND; 2 Oral and Maxillofacial Surgery, Sharad Pawar Dental College and Hospital, Datta Meghe Institute of Higher Education and Research, Wardha, IND; 3 Oral Pathology and Microbiology, Sharad Pawar Dental College and Hospital, Datta Meghe Institute of Higher Education and Research, Wardha, IND

**Keywords:** mandibular osteoma, histopathological diagnoses, radiographic findings, osteogenic tumor, peripheral osteoma, gardner's syndrome, exophytic growths, rare benign tumor

## Abstract

A benign osteogenic tumor made up of mature, well-differentiated bone tissue is called an osteoma. Jaw solitary peripheral osteomas are an uncommon occurrence. The mandible is affected more frequently than the maxilla, and the lingual side of the body, the angle, and the inferior border of the jaw are the sites of highest preference. Males are more likely than females to be impacted by osteomas, which can strike at any age. Patients with osteomas should be considered to have Gardner syndrome. This condition includes many embedded or supernumerary teeth, skeletal abnormalities such as osteoma and hyper calcification of the maxillary bones or skull, skin and soft tissue tumors, and gastroenteric polypus. Differential diagnosis is crucial since the development of gastroenteric polyps, which have a potentially malignant progression, occurs before oral and maxillofacial symptoms emerge. Mandibular osteomas are uncommon; even rarer is a massive osteoma with a prevalence of 0.01-0.04% of the population. That's the reason this is being discussed in this article. The primary differential diagnosis and pertinent clinical information from previously published literature are also included in this article.

## Introduction

A benign, slowly developing osteogenic tumor that is characterized by the growth of cancellous or compact bone is called an osteoma [1]. Osteomas of the mandible can develop in three different locations such as on the surface of the bone as a polypoid or sessile mass (periosteal or exophytic), the soft tissue (extraskeletal), or the medullary bone (central type: endosteal) [1-3]. Osteomas often only show up as isolated asymmetry or deformity, are clinically quiet, and have no symptoms. Numerous etiopathogenic theories, including congenital defects, inflammation, muscle activity, embryogenic alterations, and trauma, have been put forth to explain the genesis of osteomas. Osteomas are mostly seen in the bones of the face and neck; they are seldom, detected in other bones [4-6].

Mandibular osteomas most commonly affect young individuals, presenting as umbrella-shaped lesions with a stalk. They show up as lobulated opacities on radiography. To further classify them as a compact, cancellous, or mixed variation of peripheral osteoma, histological evidence is required. Gardner's syndrome is defined as anomalies of the teeth and skeleton in conjunction with colorectal polyposis [7,8]. Thus, pertinent research has to be conducted to assess patients in this manner. In this paper, a 50-year-old man with proliferative periostitis is described clinically. However, histological tests reveal that the patient's final diagnosis was osteoma which developed as a result of postsurgical trauma.

## Case presentation

A 50-year-old man presented himself to the Oral Medicine and Radiology Outpatient Department with the chief complaint of bony hard swelling in the lower right retromolar region of the jaw for months. The swelling was initially of a smaller size and has gradually progressed to the current size. There was no history of associated pain and discomfort caused by the swelling. The patient had undergone extraction in the lower right posterior region of the jaw six months back and since then the swelling started gradually increasing. The patient gave no history of pus discharge or bleeding from the swelling. The patient did not give any history of hot or cold fomentation. There was no relevant medical history. The patient did not have any deleterious or parafunctional habits. No history of similar presentations in any other family members. The patient was not allergic to any medications known to her to date. On extraoral examination, no gross head-and-neck asymmetry, discolorations, lesions, or masses were observed in the head-and-neck region. Temporomandibular joint movements were bilaterally smooth and synchronous with no deviation or clicking present. On palpation of lymph nodes, no regional lymphadenopathy was present.

**Figure 1:**
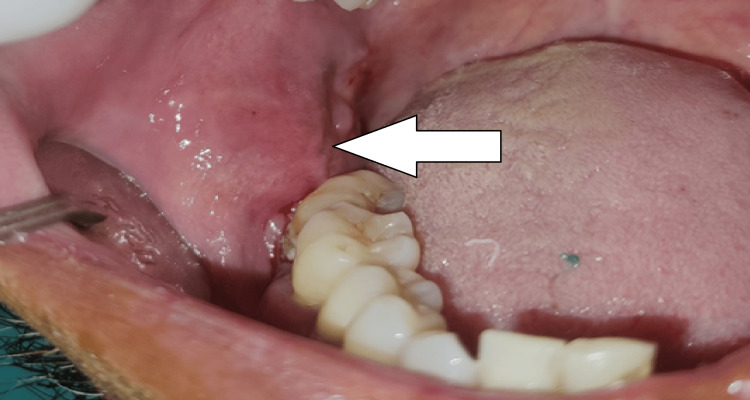
A diffuse, oval swelling in the right retromolar region of size 2 x 1 cm approximately, with an elevated surface. The overlying mucosa over the swelling seems intact and the color is the same as that of the adjacent mucosa

On intraoral examination, 48 was missing and a diffuse, oval swelling was seen in the right retromolar region of size 2 x 1 cm approximately, with an elevated surface. The overlying mucosa over the swelling was intact and the same as that of the adjacent mucosa. On palpation, it was a bony hard, non-fluctuant swelling that was non-tender. No bleeding or pus discharge on manipulation. Other intraoral findings revealed generalized chronic periodontitis (Figure 1).

**Figure 2:**
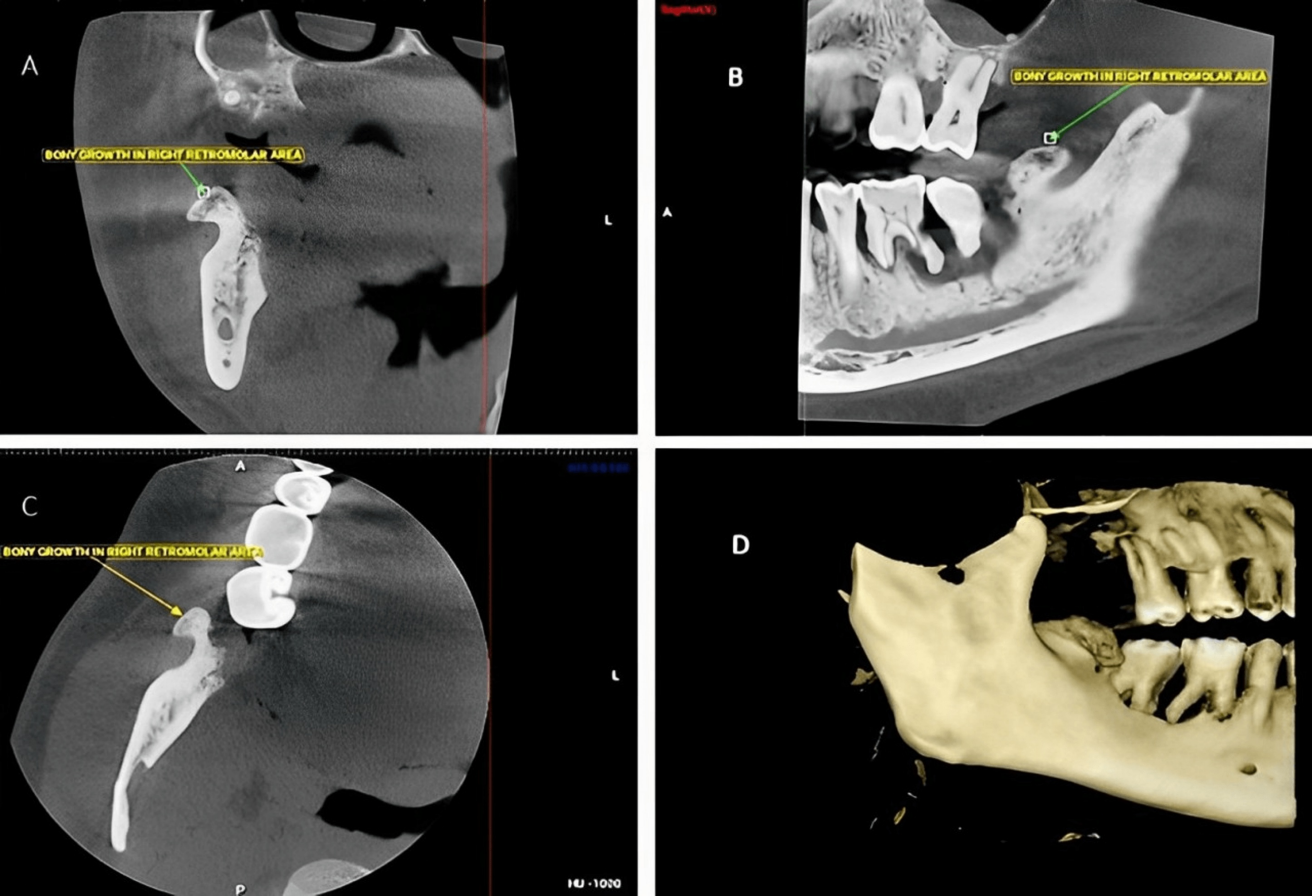
CBCT findings of the bony growth seen in the right retromolar region A: coronal view of the bony growth in the retromolar region; B: sagittal view of the bony growth in the retromolar region; C: axial view of the bony growth in the retromolar region; D: 3D reconstruction of the bony growth in the retromolar region CBCT: cone beam computed tomography

The patient was then advised for cone-beam computed tomography (CBCT) investigations which revealed bony overgrowth in the right retromolar area of size 15.9 x 6.5 x 8.8 mm, oval in shape with well-defined margins (Figure 2).

**Figure 3:**
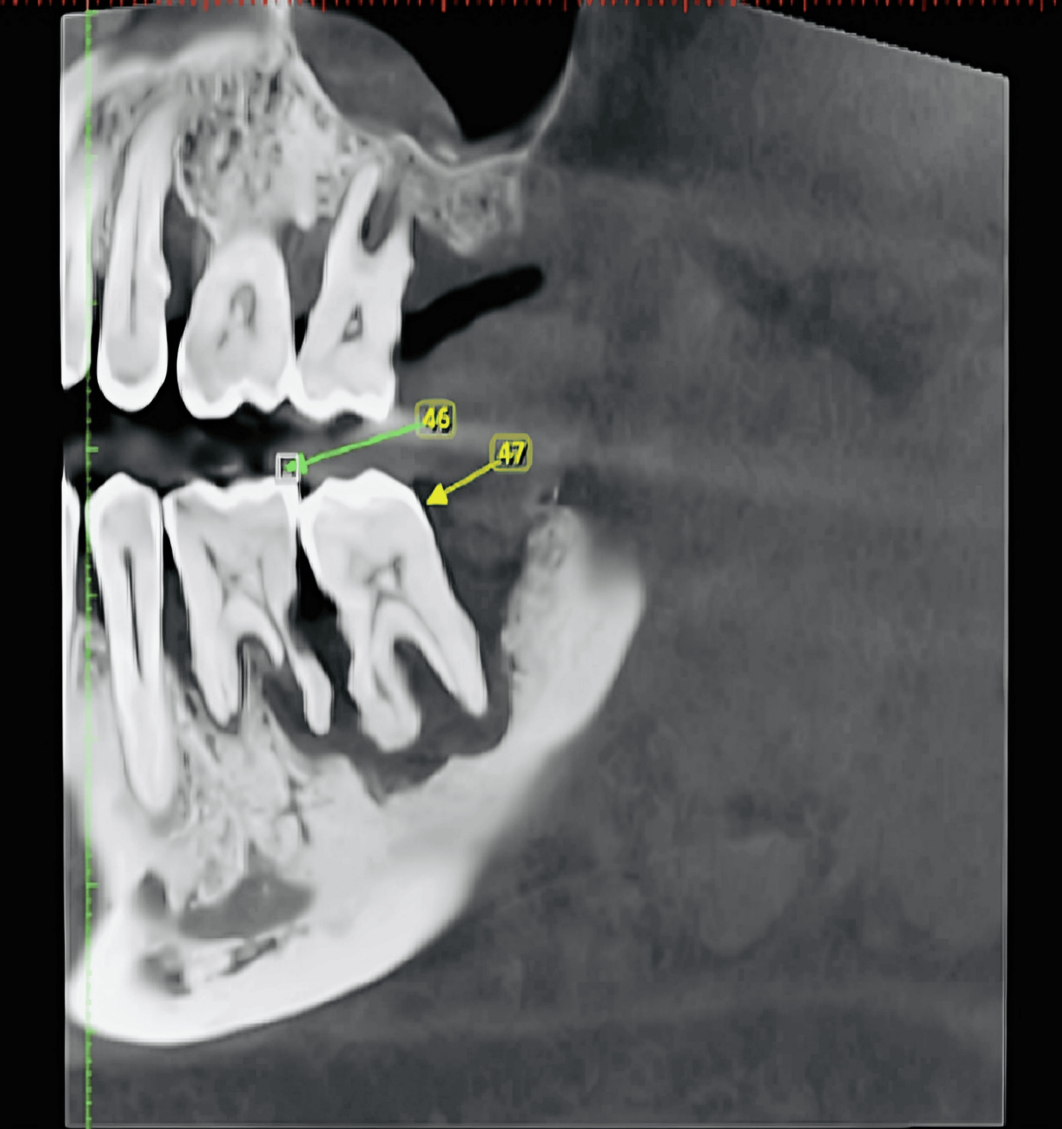
Sagittal view of CBCT revealing severe alveolar bone resorption and periapical rarefaction with 46, 47 CBCT: cone beam computed tomography

Other findings were severe alveolar bone resorption and periapical rarefaction with 46, 47 (Figure 3).

**Figure 4:**
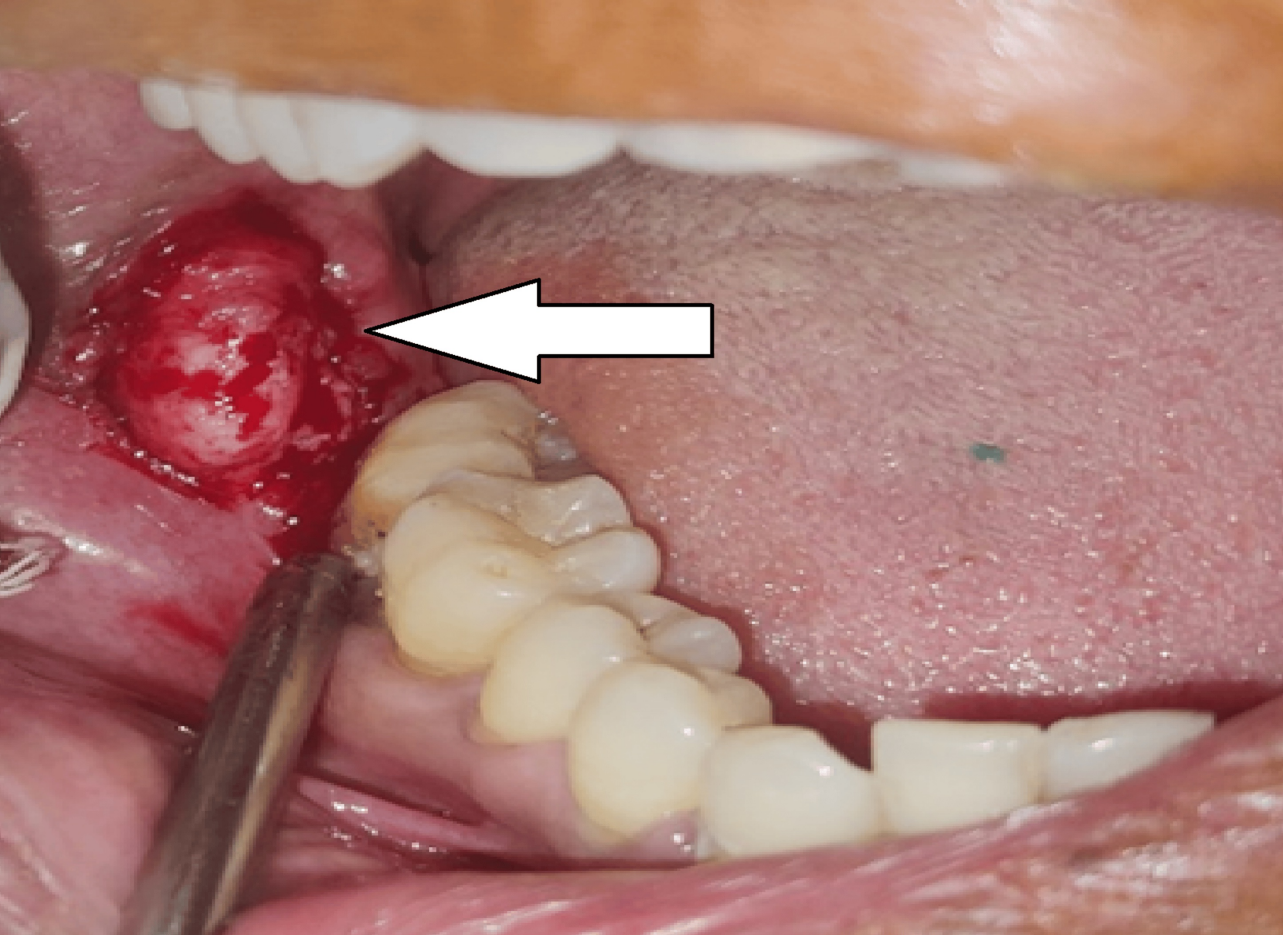
Exposed bony growth in the right retromolar region after raising the mucoperiosteal flap

Following the establishment of proliferative periostitis as a clinical diagnosis and investigating it radiographically, under local anesthesia, a biopsy of the lesion was carried out. An incision was made from the external oblique ridge on the right side of the mandible. A mucoperiosteal flap lateral to the right mandibular molar region was raised to expose the bony protuberance. It was determined to be a newly established bone covering the mandibular cortical bone in the vicinity of the lower right third molar (Figure 4).

**Figure 5:**
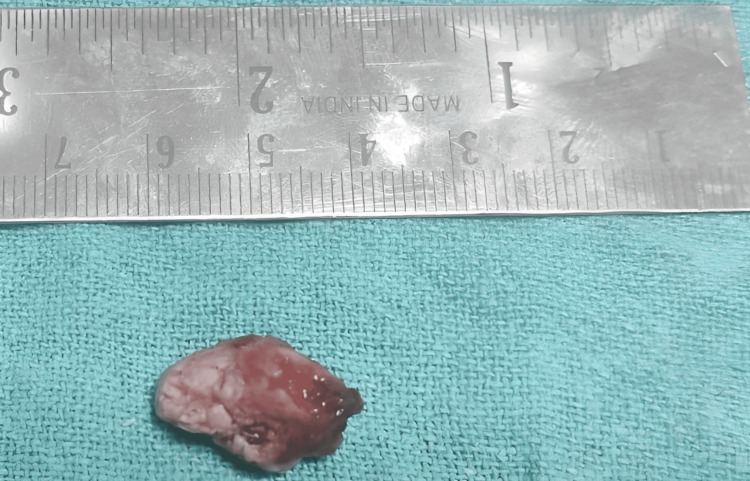
Removed specimen of the bony growth directed for histopathological investigations

Bone guttering was done using high-speed rotary instruments as well as chisel and mallet. The removed specimen was directed for histopathological investigations (Figure 5).

**Figure 6:**
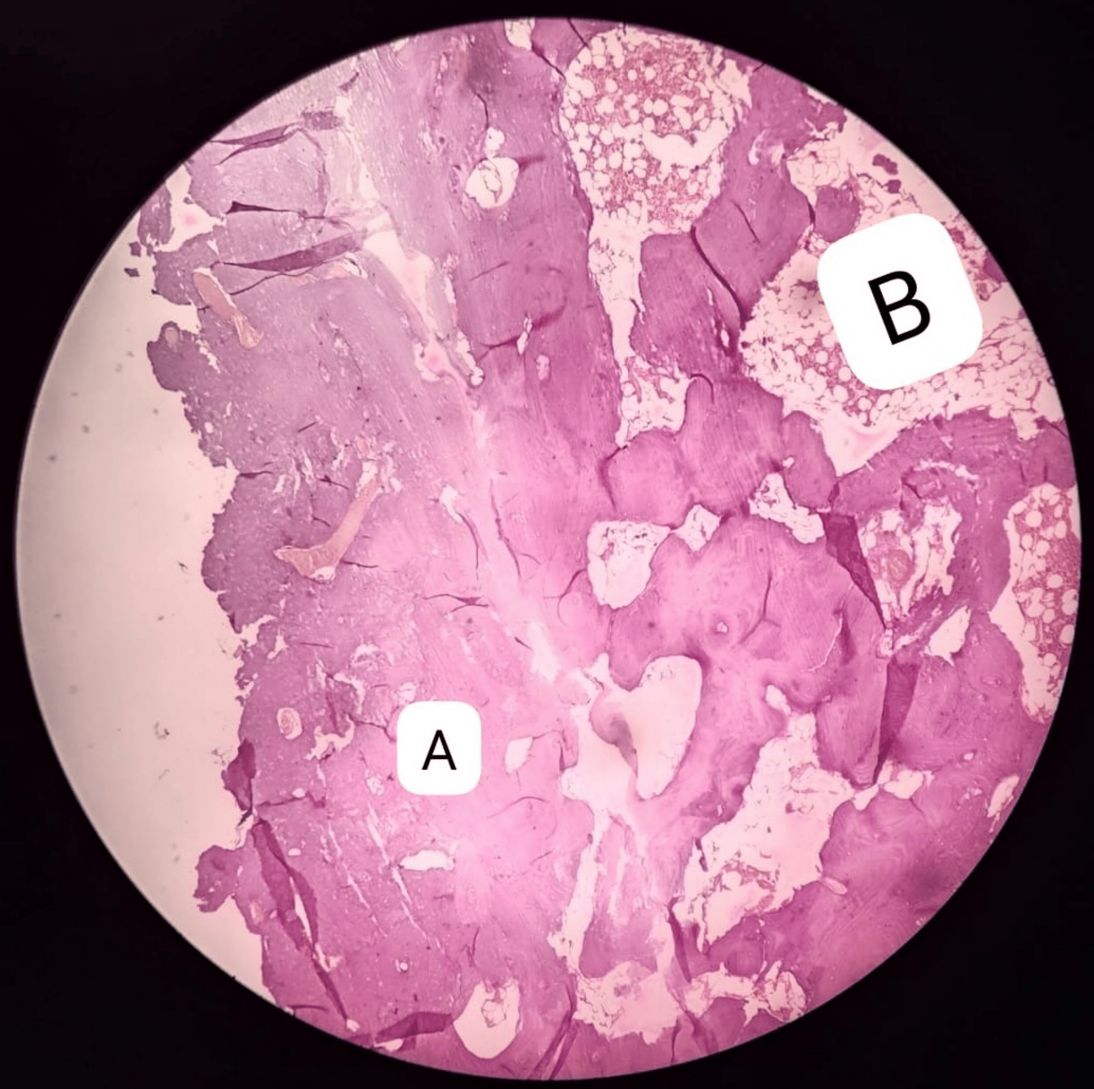
Under low power, histopathological examination of the lesional tissue shows matured osseous tissue without osteoblastic riming. The surrounding connective tissue is composed of fibrofatty tissue A: matured lamellar bone; B: fibro-fatty connective tissue

Saline irrigation was used at the operative site. A drainage tube was used to drain the wound and the wound was sutured. The histopathological examination of the tissue showed matured osseous tissue without osteoblastic riming and the surrounding connective tissue composed of fibro-fatty tissue (Figure 6).

After correlating the clinico-radio-histopathological features, the final diagnosis of the case was peripheral osteoma. Postoperatively, antibiotic treatment with amoxicillin 625 mg was continued for one week after the surgical exploration. Two weeks after the surgery, he had no signs of inflammation in the retromolar region. The patient has been followed for three months, without any evidence of recurrences.

## Discussion

Osteomas are mostly tumors of the craniofacial bone; they seldom impact the extragnathic skeleton, however, there have been documented examples of soft tissue osteomas originating inside the majority of skeletal muscles. Jaw bone osteomas are uncommon. It is stated that the majority of osteomas in this area have happened as peripheral types in the jaw bones, which is quite uncommon [9]. The lingual sides of the premolars, the inferior border of the condyle, and the inferior and lateral region of the mandibular angle are the most often involved areas in cases of mandibular involvement. Males are impacted at a ratio of around 2:1 more often than females. Osteoma affects 0.01-0.04% of the population generally, which is a low occurrence. About 2.1% of benign bone tumors and 2.9% of all bone tumors are osteomas [5]. Peripheral osteomas have unclear etiologies; however, three ideas have been proposed namely developmental, malignant, and reactive. Given that the majority of occurrences are seen in individuals who are no longer in their formative growth phase, the hypothesis that a peripheral osteoma represents a growth aberration appears unlikely [10-12]. The incredibly sluggish development is inconsistent with the neoplastic idea. When trauma is verified, the reactive explanation is more likely to be true.

Gardner's syndrome should be assessed in patients with osteomas. Adenomatoid gastrointestinal polyps, multiple osteomas, soft tissue and skin tumors, and supernumerary teeth are the components of Gardner's syndrome. These gastrointestinal polyps typically develop into cancer. Because osteomas can be seen in the early stages of Gardner's syndrome, diagnosing them is crucial and may even save lives. Dentists may also be key players in the diagnosis of colonic polyposis [12].

The size, location, and development direction of the lesion determine the clinical indications, symptoms, and consequences. Directly associated osteoma symptoms typically result from a "mass effect" when the disease compresses against normal tissues [13]. Clinically, they have a circumscribed appearance, are often spherical and protuberant, and develop extremely slowly and continuously. Osteomas are typically asymptomatic and go undiagnosed until they cause functional impairment or facial asymmetry, or until they are inadvertently discovered during a routine radiography examination. Nonetheless, headaches, facial asymmetry, occlusal dysfunction, and restricted mandibular motions may manifest, primarily in condylar osteomas, contingent on the location [5]. Even though 80% of peripheral maxillary osteomas are found in the alveolar process, mastication problems are seldom caused by them. Peripheral osteomas are identified radiographically by the presence of a well-circumscribed, radio-opaque, and oval mass that is connected to the afflicted cortical bone by a wide bass or pedicle. When considering lesion excision, a computed tomography (CT) scan, in particular a 3D reconstruction scan, is helpful in precisely describing the extent of the tumor and locating the lesion in relation to surrounding anatomical structures [1].

Three distinct forms of osteomas may be distinguished histologically such as compact, spongy, and mixed. Differentiating between an osteoma and the more frequent exostosis is necessary when an exophytic lesion appears inside the mouth cavity, yet firmly anchored to the underlying bone and with a bony consistency [6]. Although there are no histologic differences, precise case history and clinical features can help differentiate a peripheral osteoma from an exostosis. Exostosis is a bony growth in the maxillary bones' lingual plate that is often symmetric, well-circumscribed, linked to traumatic or inflammatory events, and has a restricted development pattern. It is not associated with benign neoplasia in any way. Therefore, the word "osteoma" is only used to describe lesions that exhibit benign tumor features and autonomous development [9]. Osteoid osteoma, periosteal osteoblastoma, exostoses, and peripheral ossifying fibroma are among the differential diagnoses [14]. Osteomas can continue to grow beyond adolescence; however, exostoses, which are bony excrescences thought to be hamartomas, are often observed in the hard palate (torus palatinus) and buccal and lingual regions (torus mandibularis). Osteoid osteoma, periosteal osteoblastoma, and peripheral ossifying fibroma are painful, rapidly growing, reactive localized overgrowths that often affect young individuals and are uncommon in the maxillofacial areas. Osteoma monogenicity and appearance are easily characterized and diagnosed [12]. Compared to the maxilla, the mandible is where osteomas are more common. A mandibular condyle osteoma may result in a gradual change in the patient's occlusion, with a midline deviation toward the unaffected side. In their study, Sayan et al. reported 22.8% occurrence in the mandible, whereas Kaplan et al. and Woldenberg et al. found 81.3% and 64% incidence in the mandible and 14.28% occurrence in the maxilla, respectively [8,10,15].

It is recommended to treat the osteoma by completely removing it surgically from the base where it connects with the cortical bone. No cases of osteomas becoming cancerous have been documented. Bosshardt et al. have only documented one incidence of recurrence yet [5]. It is fundamental to have routine clinical and radiological follow-ups following the surgical removal of a peripheral osteoma.

## Conclusions

In conclusion, osteomas primarily affect the maxillofacial area and manifest as lobulated, slowly developing, well-circumscribed masses. A patient seeks therapy when they have a functional disability or cosmetic deformity. A cautious oral physician would, therefore, be expected to aggressively rule out the potential of Gardner's syndrome, as the initial indication of the illness might be a benign osteoma, which eventually presents as cancerous polyps in the gastrointestinal tract.
